# Supplementary material for: Analysis of divergent gene expression between HPV + and HPV- head and neck squamous cell carcinoma patients
Source: Infect Agent Cancer. 2025 May 21;20:31. doi: 10.1186/s13027-025-00663-1 (PMC12096591; doi:10.1186/s13027-025-00663-1)
Supplement: Supplementary file 1 — Supplementary Material 1 [file 13027_2025_663_MOESM1_ESM.pdf]

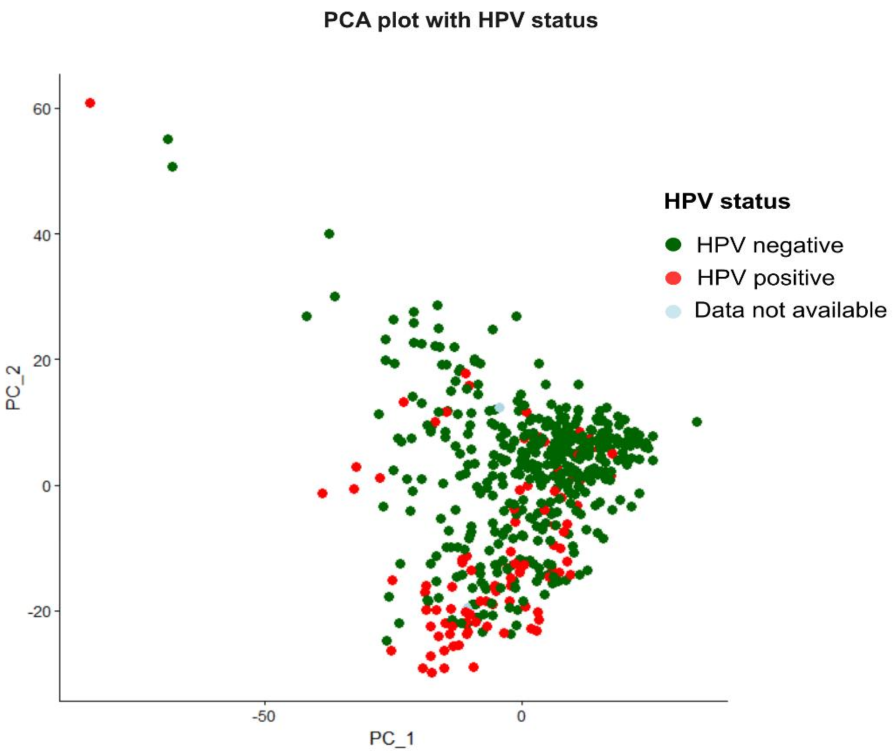

Supplementary Fig. 1- Plot showing the distribution of HNSCC patients colored on their HPV status

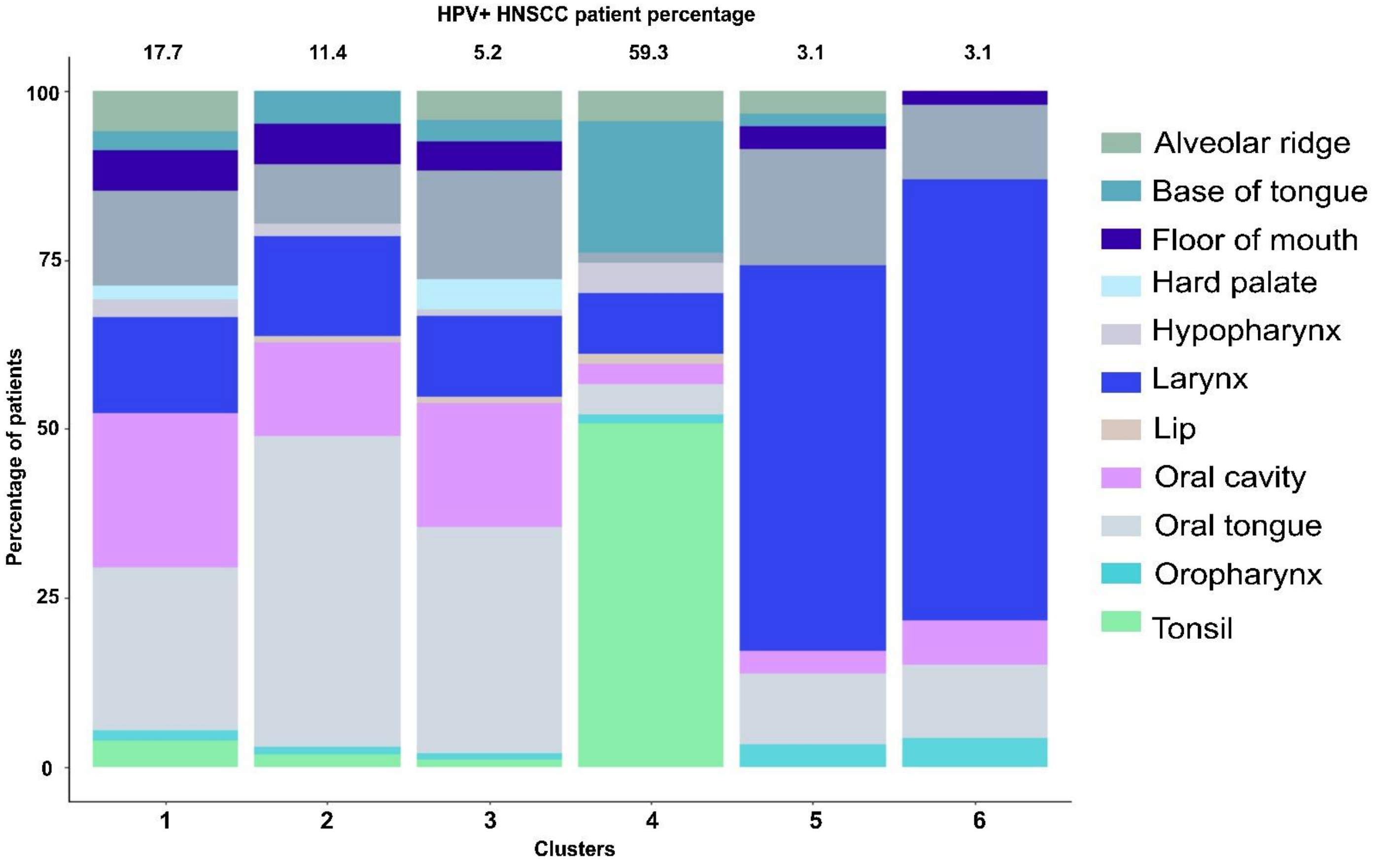

Supplementary Fig. 2- Stacked bar plot showing the distribution of the region of the patient's neoplasm in each cluster. The percentage of HPV patients in each cluster is mentioned on top of each bar.

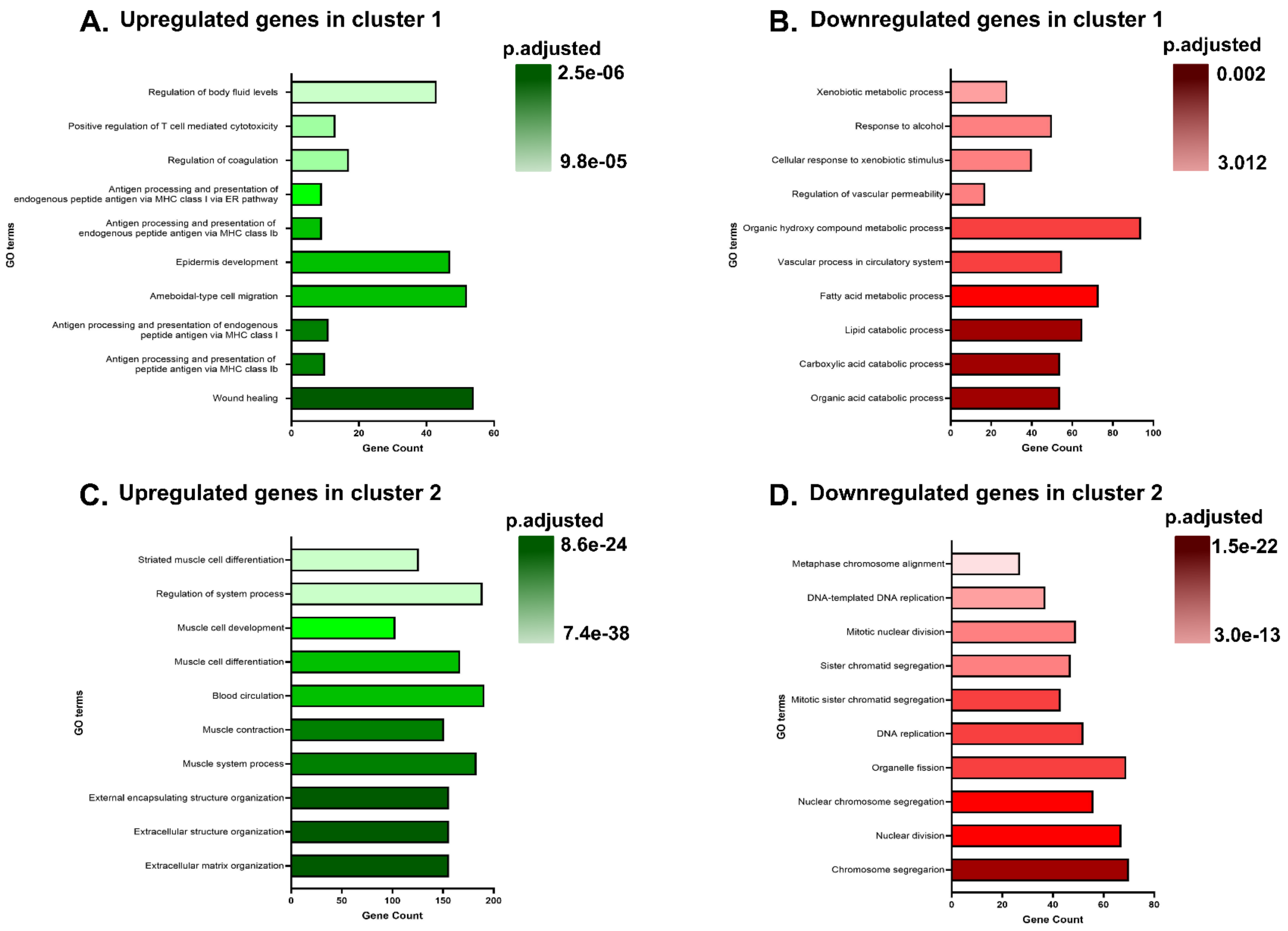

Supplementary Fig.3 Plot showing the upregulated and downregulated biological processes (BP) and the corresponding transcript counts for the clusters 1 and 2

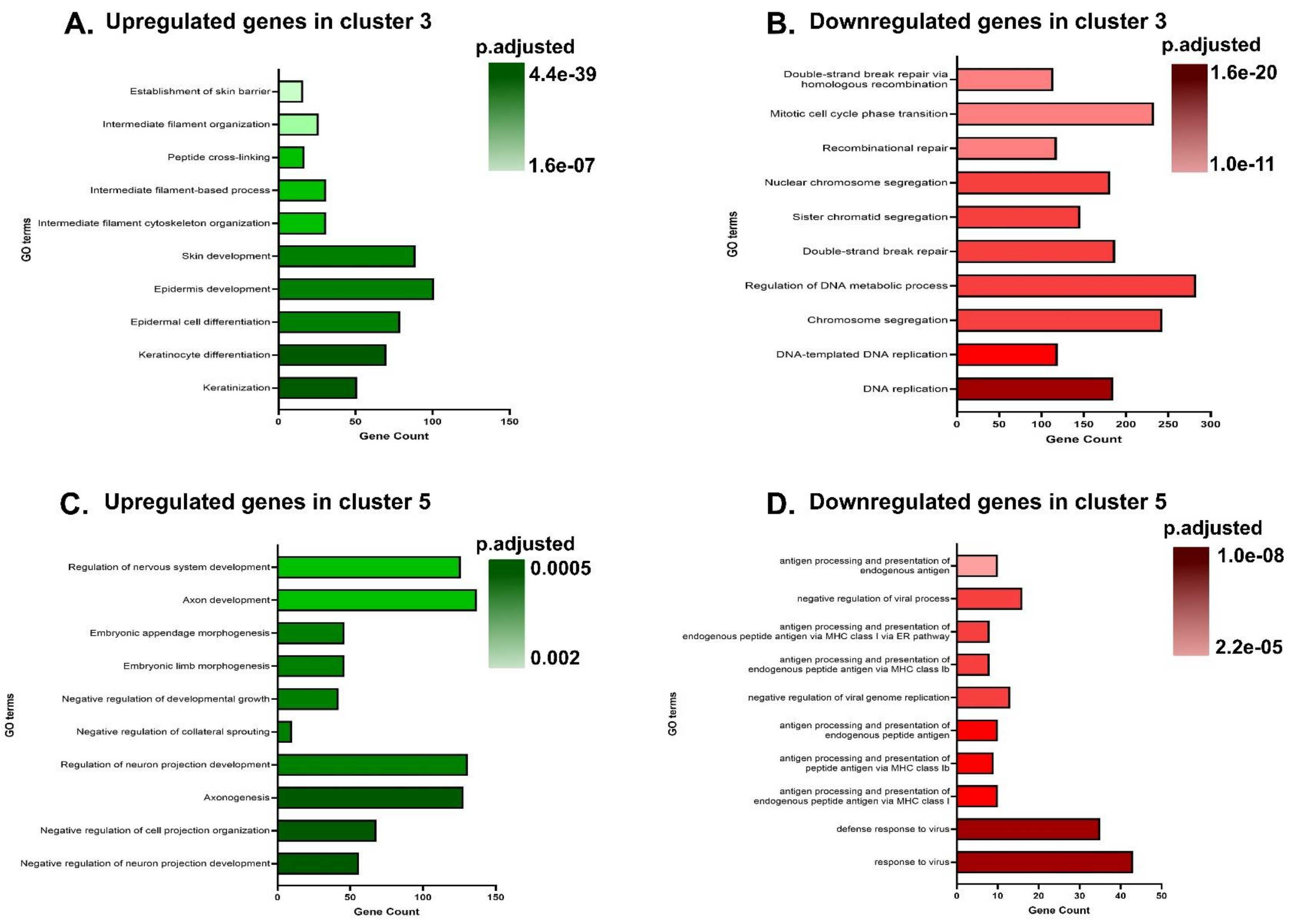

Supplementary Fig.4 Plot showing the upregulated and downregulated biological processes (BP) and the corresponding transcript counts for the clusters 3 and 5

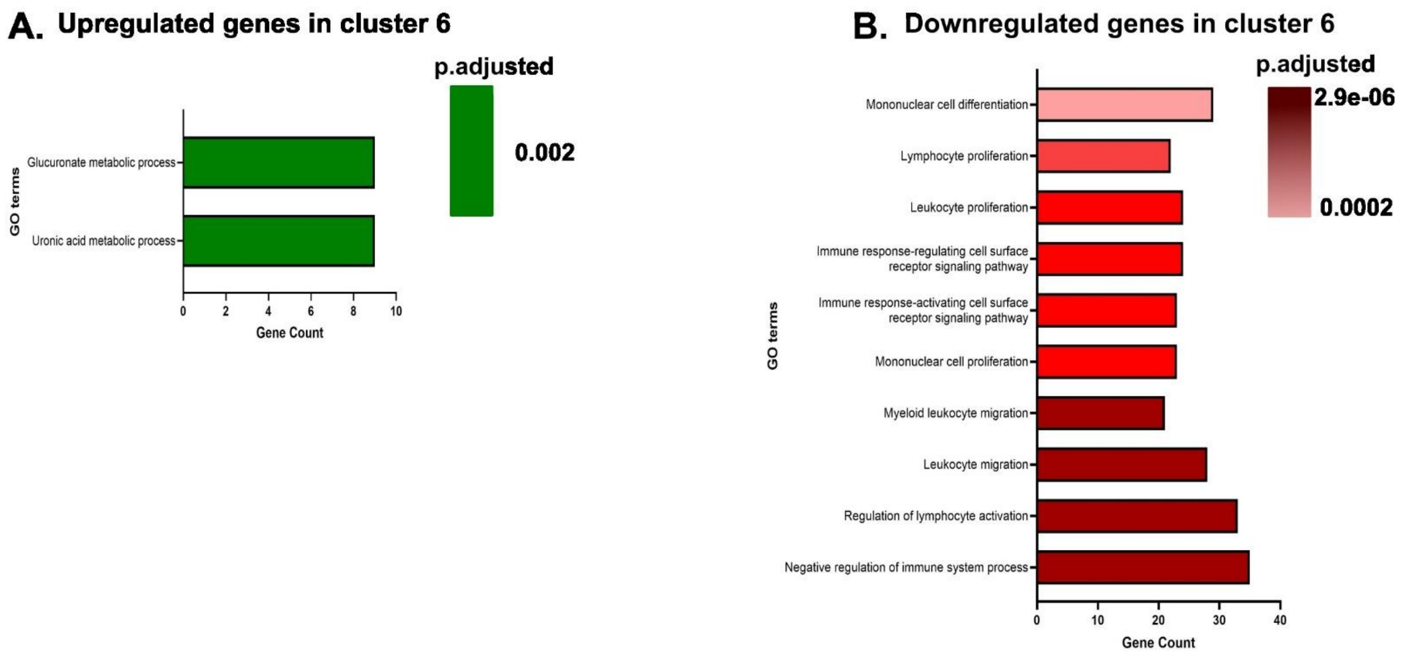

Supplementary Fig.5 Plot showing the upregulated and downregulated biological processes (BP) and the corresponding transcript counts for the cluster 6
